# Incidental findings related to genes associated to HAE-nC1INH: how to proceed?

**DOI:** 10.3389/fimmu.2025.1605727

**Published:** 2025-06-17

**Authors:** Anastasios E. Germenis, Despina Sanoudou

**Affiliations:** ^1^ Department of Immunology & Histocombatibility, School of Medicine, University of Thessaly, Larissa, Greece; ^2^ Clinical Genomics and Pharmacogenomics Unit, 4th Department of Internal Medicine, Medical School, National and Kapodistrian University of Athens, and Biomedical Research Foundation of the Academy of Athens, Athens, Greece

**Keywords:** hereditary angioedema, incidental findings, incidental VUS, penetrance, secondary findings, variants of uncertain significance

## Abstract

In contrast to hereditary angioedema (HAE) due to C1-inhibitor deficiency, the detection of pathogenic variants in genes linked to HAE with normal C1 inhibitor levels (HAE-nC1INH) is required for the diagnosis of the corresponding types of the disease. The mainstreaming of genomic technology and the increasing use of next generation sequencing have increased the possibility of an unintentional detection of HAE-nC1INH pathogenic variants and allowed the incidental finding of variants of uncertain significance (VUS) in the relevant genes. Apart from *F12* and *PLG* pathogenic variants, the current level of evidence on the prevalence and penetrance of variants associated with HAE-nC1INH does not support the reporting of their incidental finding. On the other hand, although VUS should not be used in clinical decision-making, further consideration is warranted (a) for VUS found in exon 9 of the *F12* gene after a diagnostic genetic analysis of individuals either with or without personal or family history of angioedema, and (b) for VUS found in any of the other genes linked to HAE-nC1INH, after genetic analysis performed in the context of differential diagnosis of angioedema cases. Given the complexity of interpreting, reporting and communicating incidental findings, a close partnership between patients, clinicians, laboratory geneticists and genetic counsellors is essential to optimize the management of these results.

## 1 Introduction

Until the turn of the century, HAE-C1INH was the only identified form of HAE, known to be caused by alterations in the *SERPING1* gene. Nevertheless, genotyping was not required for its diagnosis as plasma levels of antigenic or functional C1 inhibitor is a reliable diagnostic biomarker (1). Since then, pathogenic variants in eight different genes (*F12, PLG, ANGPT1, KNG1, MYOF, HS3ST6*, *CPN1* and *DAB2IP*) have been recognized as associated with a form of HAE with normal C1 inhibitor levels (HAE-nC1INH). However, in a proportion of patients with this form of HAE, classified as HAE of unknown cause (HAE-UNK), no pathogenic variant has been identified (2). It is, therefore, highly likely that additional pathogenic variants remain to be discovered. As no biochemical diagnostic biomarkers have yet been identified for HAE-nC1INH, genetic testing is required to diagnose at least its types with known genetic cause. In addition, the detection of these variants could be useful in the differential diagnosis of other forms of recurrent angioedema without hives, presenting with normal C1 inhibitor levels but lacking a known pathogenic variant, mainly mast cell-mediated and medication-associated angioedema.

The possibility of simultaneous detection of all the HAE-nC1INH causal variants using genome sequencing technologies, mainly exome or genome sequencing (WES or WGS), will expedite the diagnosis of the disease, but will also allow the incidental finding of variants of uncertain significance (VUS) in the relevant genes. At the same time, the mainstreaming of genetic testing and increasing use of next generation sequencing (NGS) increases the possibility of an unintentional detection of HAE-nC1INH pathogenic variants. It is clear that both cases present challenges that have not been previously faced by clinicians dealing with angioedema. On the one hand, even though VUS should not be considered sufficient grounds for clinical decision-making (3), the role of the clinician in elucidating the gaps in genetic knowledge highlighted by their detection could be crucial. On the other hand, the unintentional identification of a HAE-nC1INH pathogenic variant offers the chance to identify and manage a type of the disease that may otherwise be unrecognized in an individual. In these cases, however, clinicians confront the challenge of managing the disease risk associated with the incidental finding of an HAE-nC1INH pathogenic variant, which possesses a positive predictive value that is clearly lower than that of an indication-based testing result.

The various policy documents that have been published on the reporting of incidental findings and the management of the aforementioned challenges differ on fundamental issues. Although none has been accepted as a general standard, most of them state that unsolicited findings should be disclosed if they have high predictive value and are indicative of serious health problems that allow for treatment or prevention. As the angioedema community is entering the era of genomic diagnosis, it is essential to develop a policy for the management of incidental findings that could help early diagnosis of a potentially life-threatening disease with unpredictable clinical course, like HAE-nC1INH. Any policy adopted now will inevitably change as knowledge about the known pathogenic variants accumulates (prevalence, penetrance, expressivity, etc.) and as new genes involved in the pathogenesis of HAE-nC1INH are discovered.

## 2 Incidental finding of a HAE-nC1INH pathogenic variant

The spectrum of reportable incidental findings remains yet unresolved (4). The American College of Medical Genetics and Genomics (ACMG), in an attempt to address the obligations of laboratories to report findings that are not directly related to the reason for testing but are deemed medically actionable, has adopted a distinction between incidental and secondary findings that first appeared in the literature in 2012 (5). Incidental findings refer to unexpected results that arise during the genetic analysis and are unrelated to the initial diagnostic indication for testing. Variants identified incidentally, may be classified as pathogenic, likely pathogenic, VUS, likely benign, or benign. Secondary findings, on the other hand, are genomic variants that are unrelated to the primary reason for testing but deliberately sought after analysis based of a predefined list of actionable genes. The most recent ACMG recommendations specify a total of 78 genes that are to be reported as secondary findings and suggest that the list should be updated annually (6).

The criteria for the inclusion of a secondary finding in the list of ACMG are multifaceted. One of the primary criteria is the concept of clinical actionability, which refers to the ability to provide effective management based on the identified genetic variant, including treatment options that can significantly alter the clinical outcome for the patient or his/her family members. The ACMG also selects genes based on the technical feasibility of detecting variants in them, only including genes in which disease-causing variants can be easily identified from sequencing data without an undue analytic burden. Another critical criterion is the penetrance of the associated disease. Genes associated with diseases that have a high penetrance are prioritized to ensure that the identification of a secondary finding is likely to have a significant impact on the patient’s health. The ACMG also considers the quality of evidence supporting the association between the variant and the disease and emphasizes the need for a robust body of literature supporting the pathogenicity of the variant and its clinical relevance (7).

Not unexpectedly, genes associated with HAE-nC1INH are not included in the ACMG list for reporting secondary findings. However, given the clinical actionability following HAE diagnosis, this is not a barrier to reporting the unintentional identification of a HAE-nC1INH pathogenic variant. Rather, it must be seriously considered that reported cases of most of HAE-nC1INH types to date have been successfully treated with existing drugs for other forms of HAE, which present with low burden and risk while, generally, protect patients from life-threatening attacks and significantly improve their quality of life.

Nevertheless, the decision to report the incidental finding of a HAE-nC1INH pathogenic variant in an individual with no previously recognized personal or family history of the disease must take into account the incomplete penetrance of all its types. To this point, it must be underlined that the penetrance observed from family ascertainment-based studies may be less than population-based penetrance, i.e., penetrance estimated from a sample unbiased in terms of disease presence (ascertainment bias) (8). It should also be emphasized that an incidental finding constitutes a form of screening and therefore has a lower positive predictive value than an indication-based test result (9).

**Figure 1:**
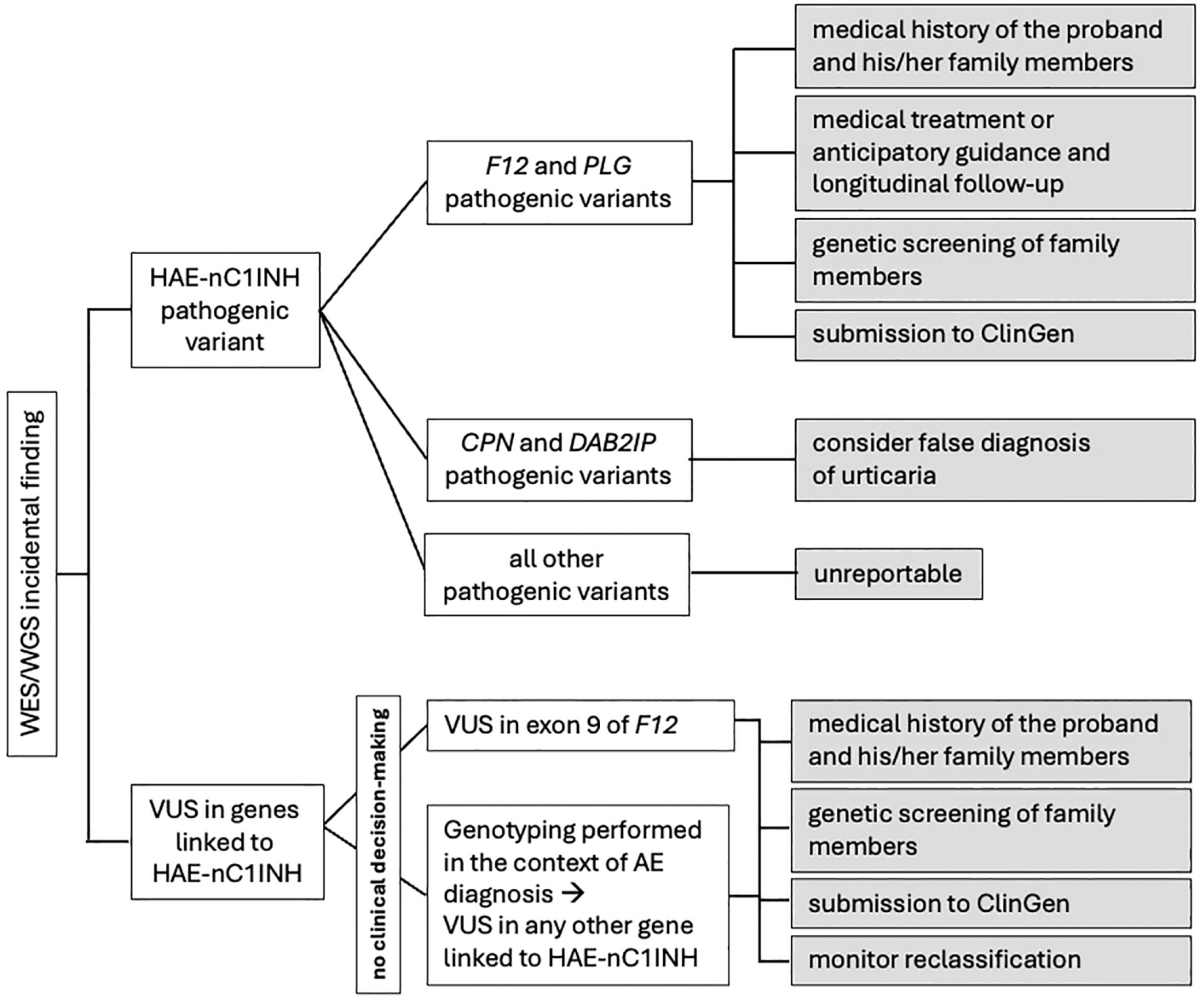
Proposed algorithm for the management of incidental findings related to genes associated to HAE-nC1INH.

Apart from HAE-FXII and HAE-PLG, penetrance estimates for all other types of HAE-nC1INH are based on the study of the families in which the disease was first recognized. Therefore, reporting of the unsolicited finding of these six HAE-nC1INH pathogenic variants will not be justifiable until reliable estimates of their penetrance and expressivity have been collected. A possible exception would be the incidental finding of pathogenic variants in the *CPN* and *DAB2IP* genes. In both cases, reported patients suffer from HAE with hives (10, 11). Therefore, it is advisable to examine the medical history of the probands for a false diagnosis of urticaria. Regardless, incidental findings detected in all the eight genes associated with HAE-C1INH need to be reported in public databases (see below), while clinicians must be aware of upcoming results on their penetrance, which may lead to a re-evaluation of their initial decision not to report them (**Figure 1**).

As far as HAE-FXII and HAE-PLG are concerned, despite their incomplete penetrance, existing evidence on their prevalence justifies the adoption of a management strategy that accounts for the increased disease risk associated with the relevant incidental findings. Such a strategy first of all must include complete information and a detailed medical history of the proband and his/her family members, including whether female members encountered swelling during periods of elevated estrogen, in order to reveal previously unrecognized symptoms of angioedema (e.g., abdominal symptoms attributed to other conditions, swelling attacks attributed to antihypertensive drugs, unnecessary surgical procedures, etc.). Obviously, in the absence of overt clinical disease medical treatment is not justified but anticipatory guidance as well as longitudinal follow-up of carriers is necessary. Thereafter, genetic screening should be done on family members, irrespective of whether or not they have experienced unrecognized symptoms of angioedema, to detect the segregation of the pathogenic variant (12), and reporting of the variant/case in public databases (see below) must be considered.

Estimating the disease risk associated with the unsolicited finding of pathogenic variants, and therefore formulating an appropriate management strategy, is more complicated in the case of HAE-FXII. This is due to the fact that more than one *F12* variants have been found to be associated with the disease (13), as well as to ethnic differences observed in disease prevalence. The disease risk could also be affected by the prevalence of incidental findings in WES and WGS studies, which are known to vary between countries, but unfortunately this parameter is unknown in Brazil, where the highest prevalence of HAE-FXII has been reported (14).

## 3 Incidental VUS in genes already linked to HAE-nC1INH

Variants with insufficient or conflicting evidence supporting disease association, such that they cannot be classified as either likely pathogenic or benign, according to the ACMG guidelines, are classified as VUS (3). Although, according to the same guidelines, VUS should not be used in clinical decision-making, consistent policies about reporting and interpreting of VUS have not yet been fully debated or established. As a result, in practice, VUS lead to patient and provider confusion, are often misinterpreted as significant positive findings, and do alter clinical management especially when non-specialists guide patients regarding genetic information. Consequently, early information of angioedema experts will help to avoid such problems in HAE-nC1INH patients.

Generally, VUS substantially outnumber pathogenic findings. More specifically, up to now, near a thousand of VUS located in the eight genes linked to HAE-nC1INH have been submitted to ClinVar, one quarter of them in the *PLG* gene. Interestingly, 78 of these VUS have been detected during genetic testing of HAE patients. VUS, however, differ greatly in their likelihood of being pathogenic. Only a minority of them, described as “hot/warm VUS”, are likely to be reclassified as (likely) pathogenic when additional proof of pathogenicity is obtained, and these are the only ones that are typically considered for reporting back to the ordering clinician (15). But how can “hot VUS” be recognized?

The approach to interpretation of an incidentally found VUS is complex and demands consideration of several factors which may indicate pathogenicity, including (a) the frequency at which the variant is found in the general population, (b) the prior observation of the variant in other individuals with similar phenotype, (c) the localization of the variant to key protein domains, and (d) the high evolutionary conservation of the affected amino acid across species, among others. The challenge of interpreting VUS is compounded by the fact that most variations identified and interpreted in rare disease testing are unique to an individual and may never be seen again. Indeed, 78% of variants submitted to ClinVar have only been submitted by one laboratory, primarily because they have been observed only once in a single individual (16). Analysis of the Genome Aggregation Database (gnomAD) dataset suggests that every individual harbors on average 27 unique and 200 very rare variants in their coding sequencing alone (17). It can thus be concluded that the interpretation of “hot VUS” is a demanding and time-consuming process possibly involving a multidisciplinary setting (i.e. with the genetic laboratory, the referring clinician and the patient). Therefore, its implementation towards classifying a VUS as “hot”, is not feasible without clinical judgement considering the pre-test probability of each case pathogenicity.

Bearing the above in mind and given the current level of evidence on the genetics of HAE-nC1INH, VUS unintentionally found in genes of uncertain significance can by no means be considered “hot”. On the contrary, it is suggested that incidental VUS found in genes associated with HAE-nC1INH could be considered appropriate for reporting and interpreting in two cases. Firstly, as all the four HAE-nC1INH pathogenic variants are located in exon 9 of the *F12* gene, VUS detected in this particular exon, following the diagnostic genetic analysis of individuals either with or without personal or family history of angioedema, deserve careful consideration. Similarly, VUS found in any of the other genes linked to HAE-nC1INH, after genetic analysis performed in the context of differential diagnosis of angioedema cases, may also warrant consideration.

It is clear that the incidental detection of the above-mentioned VUS does not contribute to the diagnostic decision. The referring clinician, however, can play a decisive role towards the clarification of the clinical significance of these variants (**Figure 1**). Firstly, VUS must be reported to Clinical Genome Resource (ClinGen) (18). Reporting VUS in ClinGen, along with as much data as possible to enhance the quality of their curation, allows them to be systematically compiled, increasing the likelihood of their reclassification over time. Secondly, genetic screening of family members could provide information about the penetrance of the variant as well as help with its interpretation. A *de novo* VUS (not inherited from either parent) has a higher likelihood of being likely pathogenic, while a VUS found to be inherited from an unaffected parent is less likely to be pathogenic (19, 20). Even after parental testing, however, variants often remain VUS. Finally, it is important that clinicians understand that the classification of a variant as VUS dependent upon scientific knowledge at the time of the assessment and that new information generated subsequently may change its classification. It appears that advances in data sharing, computational tools, functional studies, and clinical evidence will support the prediction that VUS in coding regions will be resolved by 2030 (16). It is therefore recommended that diagnostic labs monitor the reclassification of VUS they identified through the various resources available, such as following ClinVar alerts or ClinGen updates, and inform the ordering clinicians and/or the patients, accordingly. At this point, though, it must be emphasized that VUS are more often classified as benign or likely benign than pathogenic or likely pathogenic, as well as that there are no robust predictors to identify the probability that a VUS will be reclassified when it is first detected (21).

## 4 Communicating incidental findings to the patient

The decision regarding communication of incidental findings to the patient should be determined before ordering the test, during an extensive informed consent process. However, preparing patients for the possibility of unintentionally finding an uncertain indication of a potentially life-threatening disease, like HAE-nC1INH, or for a potential VUS result is challenging.

The detection of incidental findings in genetic testing often elicits complex emotional responses. Incidental findings can trigger anxiety, decisional regret, or proactive health management, depending on individual perceptions, interpretations and personality. Those viewing genetic data as empowering embrace incidental findings as opportunities for prevention, while others experience distress over uncertain risks. Cognitive appraisal also plays a critical role. Individuals who framed incidental findings as manageable through lifestyle changes or surveillance reported lower long-term distress compared to those perceiving the findings as existential threats (22).

In these settings the role of genetic counsellors can be highly impactful. Although the establishment of this specialty is lagging in some countries, the increasing frequency of genetic testing and complexity of test results are rapidly rendering it a necessity. Genetic counsellors have a multifaceted role. At the pre-testing stage they are tasked with the responsibility of educating patients about basic genetic concepts, discussing test options, procedures, benefits and limitations. Furthermore, they prepare patients for the different types of information that may emerge from the selected genetic test, including incidental findings and VUS, along with the approaches for handling this information should it arise. The potential implications of genetic test results for other family members are also, best introduced at this stage. The overall goal of pre-testing counselling is presenting genetic testing solutions to open clinical questions, nurturing realistic expectations, raising awareness of the range of possible outcomes and minimizing pre-test related anxiety.

At the post-testing stage, genetic counsellors facilitate communication between genetic labs, clinicians and patients. Especially in the context of incidental findings, post-test counselling offers additional education and discussion of the meaning of the genetic results, and is required to mitigate subjective or mis-interpretations, unrealistic views or decisional regret. Tailored support is provided to patients and their family members, as needed, to process the new information and come to terms with the findings. Patients often need assistance in placing the impact of genetic findings in perspective and appreciating its true magnitude. The majority of patients prefer to know everything. However, the communication of genetic results needs to be tailored to their health literacy and background, beliefs, needs (physical and emotional), wishes, and psychological status at the given time. Empathetic, respectful and non-directive communication is integral to effectively supporting patients towards, often difficult yet critical, decision-making steps and lifestyle changes. Following genetic counseling, patients need to be referred directly back to the ordering clinician for medical management recommendations based on the results (23). Longitudinal studies reveal that most individuals adapt to incidental findings over time, with minimal lasting negative impact.

Incidental findings often carry implications for family members, which can be especially challenging for those who were not consulted and did not consent. Since healthcare professionals cannot initiate communication with family members, appropriate guidance and support to the patient is needed to ensure this highly sensitive information is communicated by them in a delicate yet effective manner, to maximize health benefits for their family, while minimizing complications and distress (23).

Given the complexity of interpreting, reporting and communicating incidental findings, a close partnership between patients, clinicians, laboratory geneticists and genetic counsellors is essential to optimize the management of these results and the implementation of next steps.
